# *In vitro* evaluation of ammoniation–fungal fermentation of citronella straw: Impacts on digestibility, ruminal fermentation, and palatability in Indonesian native sheep

**DOI:** 10.14202/vetworld.2025.3094-3108

**Published:** 2025-10-20

**Authors:** Dicky Pamungkas, Yenni Yusriani, Solehudin Solehudin, Gresy Eva Tresia, Mariyono Mariyono, Windu Negara, Paulus Cornelius Paat, Kiston Simanihuruk, Zul Efendi, Iman Hernaman, Budi Ayuningsih, Ade Syahrul Mubarak, Ezi Masdia Putri, Putut Suryo Negoro, Dimar Sari Wahyuni

**Affiliations:** 1 Research Center for Animal Husbandry, Research Organization for Agriculture and Food, National Research and Innovation Agency of The Republic of Indonesia, Bogor, Indonesia; 2 Department of Animal Nutrition and Feed Technology, Faculty of Animal Husbandry, Padjadjaran University, Sumedang, Indonesia

**Keywords:** ammoniation, Citronella straw, digestibility, fungal fermentation, methane mitigation, *Pleurotus ostreatus*, rumen fermentation, ruminant nutrition

## Abstract

**Background and Aim:**

Citronella straw (*Cymbopogon nardus* L.), a byproduct of essential oil extraction, is rich in lignin therefore poorly digestible, which limits its use as livestock feed. This study examined the impact of ammoniation and fungal fermentation using *Pleurotus ostreatus*, *Trichoderma harzianum*, and *Aspergillus niger* on the nutritional value, digestibility, and palatability of citronella straw for ruminants.

**Materials and Methods:**

Six treatments were evaluated *in vitro* with five replications: Ammoniated citronella straw (CsA), citronella straw fermented with *P. ostreatus* (CsFP), citronella straw fermented with *A. niger*, ammoniated and fermented with *T. harzianum* (CsAFTh), ammoniated and fermented with *P. ostreatus* (CsAFP), and ammoniated and fermented with *A. niger*. Samples were analyzed for proximate composition, fiber fractions, phenolic content, *in vitro* digestibility, and rumen fermentation parameters (pH, ammonia, volatile fatty acids, and methane). Palatability of selected treatments (CsAFTh vs. CsAFP) was tested in 18 Ettawa goats (18 months; 22.4 ± 5.5 kg).

**Results:**

The CsAFP significantly enhanced nutritive value, reducing acid detergent fiber (63.3% in CsA to 53.9%) and acid detergent lignin (15.7% in CsA to 11.4%), while increasing crude protein (9.1% vs. 6.4%–8.4%). Dry matter digestibility improved by 10%–12% (p < 0.0001). Rumen fermentation showed increased propionate, reduced acetate: propionate ratio, and CH_4_ reduction of 0.5 mmol/L. Palatability testing revealed higher voluntary feed intake for CsAFP (98 g at 360 min) compared with CsAFTh (36 g).

**Conclusion:**

CsAFP most effectively improved the nutritional quality, digestibility, and palatability of citronella straw. This strategy reduces lignin, enhances fiber utilization, shifts fermentation toward propionate, and decreases CH_4_ emission, supporting its potential as a sustainable feed for smallholder ruminant production. Further *in vivo* studies are warranted to confirm long-term performance, safety, and field applicability.

## INTRODUCTION

The utilization of agricultural byproducts as alternative feed sources offers a sustainable strategy to enhance livestock productivity while reducing environmental burdens. Citronella straw (*Cymbopogon nardus* L.), a byproduct of essential oil extraction, is one such underutilized material. Global citronella oil production is estimated at ~4,000 tons annually, primarily sourced from China and Indonesia, resulting in substantial amounts of straw waste. Field reports indicate yields of 338 kg oil/ha/year in India and up to 454 kg/ha/year in the Philippines [1, 2]. As the world’s second-largest producer after China [3], Indonesia generates considerable quantities of citronella straw, yet only ~1% of the plant biomass is utilized for oil production, leaving the majority unexploited [4]. The COVID-19 pandemic sharply reduced farmer participation in citronella production, but recovery has since begun. For smallholder farmers, treated citronella straw represents a low-cost feed option that can reduce concentrate use, improve growth or milk yield, and simultaneously minimize agricultural waste. However, adoption is hindered by limited knowledge and technical skills for processing this byproduct [5].

Although citronella straw has potential as a ruminant feed, its high fiber and lignin contents severely restrict digestibility [6]. Nutrient composition analyses show 7.72% crude protein (CP), 2.56% crude fat, 32% crude fiber (CF), 8.6% ash, 0.82% calcium (Ca), 0.19% phosphorus (P), and 46.62% total digestible nutrients (TDN) [7]. Lignin concentrations can reach up to 31% [8], which drastically limits nutrient availability [9]. Ammoniation and fungal fermentation have been proposed as methods to improve the nutritional value of lignocellulosic residues [10–12]. A previous study by Conway and O’Malley [7] demonstrated that ammoniation combined with *Trichoderma harzianum* fermentation enhanced CP and reduced fiber fractions (neutral detergent fiber [NDF], acid detergent fiber [ADF], and lignin), leading to improved digestibility and ruminal fermentation. These findings support the use of targeted chemical–biological treatments as effective tools for upgrading low-quality feed resources.

A key innovation is the sequential application of ammoniation and *Pleurotus ostreatus* fermentation. Ammoniation disrupts lignin–carbohydrate complexes, increasing fiber accessibility, while *P. ostreatus* produces oxidative enzymes that selectively degrade lignin. Together, these processes achieve greater improvements in digestibility than either treatment alone [13–15], offering a more efficient strategy for upgrading lignin-rich citronella straw.

Despite these promising advances, knowledge gaps remain. The potential of other lignocellulolytic fungi, such as *Pleurotus* and *Aspergillus*, is not fully understood. *Pleurotus* species are notable for producing lignin-degrading enzymes, including laccase (Lac) and manganese peroxidase (MnP), whereas *Aspergillus* species secrete fibrolytic enzymes that hydrolyze complex carbohydrates [16, 17]. Incorporating these fungi could further enhance lignocellulosic degradation and nutrient release [10, 18–21]. However, the interactions between fungal activity and residual essential oils in citronella straw require further investigation.

Despite the demonstrated potential of ammoniation and fungal fermentation in upgrading lignocellulosic feed resources, their combined application to citronella straw remains underexplored. Most previous studies have focused on rice straw, corn stover, and other crop residues, while systematic evaluations on citronella straw are scarce. Furthermore, the comparative performance of different fungal species, particularly *P. ostreatus*, *T. harzianum*, and *Aspergillus* niger, when integrated with ammoniation has not been thoroughly investigated. The influence of these treatments on ruminal fermentation patterns, such as volatile fatty acid (VFA) profiles and methane (CH_4_) emissions, also remains poorly documented. In addition, limited information is available on the palatability of treated citronella straw, which is a critical factor influencing voluntary feed intake and practical adoption by farmers. These gaps restrict the translation of laboratory findings into on-farm applications and hinder the development of sustainable feeding strategies for ruminants in citronella-producing regions.

Therefore, the present study was designed to systematically evaluate the effects of ammoniation–fungal fermentation of citronella straw on its chemical composition, nutrient digestibility, and ruminal fermentation characteristics under *in vitro* conditions. The study compared three fungal species with distinct lignocellulolytic capacities (*P. ostreatus*, *T. harzianum*, and *A. niger*) to identify the most effective treatment. In addition, a palatability trial was conducted using Indonesian native sheep to assess voluntary feed intake of selected treatments, thereby linking laboratory outcomes with animal preference. By integrating chemical, microbial, and animal response assessments, this research aims to provide comprehensive evidence on the feasibility of converting citronella straw into a high-quality, sustainable feed resource for smallholder ruminant production systems.

## MATERIALS AND METHODS

### Ethical approval

All experimental procedures were approved by the Animal Care and Use Commission, Agency for National Research and Innovation, Republic of Indonesia (Approval Number: 096/KE.02/SK/05/2023).

### Study period and location

The *in vitro* study was conducted from May to August 2023 at the Laboratory of the Faculty of Animal Husbandry, Padjadjaran University, Jatinangor, Sumedang, West Java, Indonesia (6°55′15′′ S, 107°46′22′′ E). A palatability trial was later conducted in communal pens housing Ettawa goats in Sumberagung Village, Grati, East Java (7°42′40′′ S, 112°59′35′′ E).

### Experimental design and treatments

Six dietary treatments of citronella straw were prepared and evaluated *in vitro*, each with five replicates:

1. CsA – Ammoniated citronella straw
2. CsFP – Citronella straw fermented with *P. ostreatus*
3. CsFAn – Citronella straw fermented with *A. niger*
4. CsAFTh – Ammoniated and fermented with *T. harzianum*
5. CsAFP – Ammoniated and fermented with *P. ostreatus*
6. CsAFAn – Ammoniated and fermented with *A. niger*

The fungal strains were obtained from the IPB Culture Collection (IPB University): *P. ostreatus* (IPB CC 15 1254), *A. niger* (IPB CC 93 0265), and *T. harzianum* (IPB CC 10 0660).

### Preparation of citronella straw

Citronella straw was collected from an essential oil refining facility in Parakansalak District, Sukabumi Regency, West Java, Indonesia. Fresh straw was selected immediately after distillation to prevent spoilage, sun-dried, and further dried under tarpaulins for 4 days. The dried straw was chopped manually into 3–5 cm lengths before treatment.

### Ammoniation procedure

Ammoniation was conducted following established protocols [3]. Urea was dissolved in water at a 1:25 ratio, applied at 4% of straw weight, and uniformly sprayed over the chopped straw. The treated straw was packed into 50 × 85 cm polyethylene bags, sealed to create anaerobic conditions, and stored at room temperature for 21 days.

### Fungal fermentation

After ammoniation, the straw was aerated to reduce ammonia (NH_3_) odor before fermentation. For inoculation, fungal cultures were prepared on potato dextrose agar (PDA). PDA was prepared from fresh potatoes (0.5 kg), water (0.5 L), sugar (200 g), agar (14 g), and chloramphenicol (2 tablets). Potato cubes were boiled, strained, and the extract mixed with sugar and agar and then sterilized and poured into Petri dishes. Fungal inocula were streaked onto PDA using sterilized loops and incubated at room temperature for 5–7 days.

For fermentation, an inoculum equivalent to 6% of straw weight was suspended in distilled water, thoroughly mixed with citronella straw, and sealed in 50 × 85 cm polyethylene bags. Anaerobic fermentation proceeded at room temperature for 8 days.

### Chemical analysis

Proximate composition (dry matter [DM], CP, ether extract (EE), ash, Ca, and P) was determined according to the Association of Official Analytical Chemists (AOAC) International methods [4]. Fiber fractions (NDF, ADF, acid detergent lignin [ADL]) were analyzed using the Van Soest method [5]. Hemicellulose was calculated as NDF–ADF and cellulose as ADF–ADL.

### Determination of total phenolic content

Total phenolic content was measured using the Folin–Ciocalteu method [22]. A 50 mg sample was incubated with Folin–Ciocalteu reagent and sodium carbonate, diluted, and the absorbance was measured at 760 nm. Standard curves were prepared using gallic acid solutions (0.2–100 ppm). Results are expressed as gallic acid equivalents (GAE).

### *In vitro* digestibility and fermentation

Digestibility was evaluated using the Tilley and Terry method [6]. Rumen fluid was collected from yearling sheep maintained on elephant grass and concentrate (60:40 ratio). The fluid was filtered through four layers of cheesecloth and flushed with carbon dioxide.

Each 125 mL flask contained 0.5 g of feed, 40 mL of buffer, and 10 mL of rumen fluid. Five replicates per treatment were incubated at 39°C for 48 h. Residues were filtered, oven-dried (105°C, 24 h), and ashed (450°C–600°C, 8 h) to determine DM and organic matter (OM) digestibility.

Supernatants were analyzed for ammonia (N–NH_3_) using the Conway microdiffusion method [7] and total VFAs using gas chromatography–mass spectrometry (Shimadzu QP 2010 Ultra with Mega-Wax-MS column). Individual VFAs (acetate, propionate, butyrate, isobutyrate, isovalerate, and valerate) were quantified.

### Statistical analysis

Data were analyzed using SAS 9.0. One-way analysis of variance was performed, and significant means were separated by Duncan’s multiple range test. A significance level of p < 0.05 was applied.

### Palatability trial

A palatability test was conducted to compare CsAFTh and CsAFP. Eighteen Ettawa goats (18 months; 22.4 ± 5.5 kg body weight) were randomly assigned to two communal pens (200 × 280 × 100 cm, raised 70 cm above ground, shaded roof). Pen 1 received CsAFTh, while Pen 2 received CsAFP. Feeders were plastic buckets, and water was provided *ad libitum*.

After a 12-h fast, feed was offered *ad libitum* for 360 min. Voluntary feed intake (VFI) was recorded at 60, 180, and 360 min as the difference between feed offered and feed refused [9]. Data were analyzed descriptively.

## RESULTS

### Chemical composition

The chemical composition of citronella straw (Table 1) varied significantly among treatments (p < 0.05 or p < 0.01), with highly significant differences (p < 0.0001) observed for DM, CP, CF, EE, TDN, Ca, P, ADF, ADL, cellulose, and hemicellulose. More modest yet significant differences were detected for OM, ash, and nitrogen-free extract (NFE) (p = 0.0229–0.0369).

**Table 1:** Chemical composition of the citronella straw (%Dry matter).

Chemical composition (%)	Treatments						SEM	p-value

	CsA	CsFP	CsFAn	CsAFTh	CsAFP	CsAFAn		
Dry matter	42.23^b^	88.79^a^	89.25^a^	41.91^b^	41.18^b^	42.31^b^	4.49	<0.0001
Organic matter	92.44^a^	91.35^ab^	92.39^a^	90.41^b^	92.05^a^	92.12^a^	0.22	0.0229
Ash	7.56^b^	8.65^ab^	7.61^b^	9.59^a^	7.95^b^	7.88^b^	0.22	0.0229
Crude protein	8.37^ab^	6.37^c^	6.74^c^	6.39^c^	9.09^a^	8.08^ab^	0.23	<0.0001
Crude fiber	30.84^bc^	33.47^a^	31.65^b^	33.91^a^	29.71^c^	30.44^bc^	0.36	<0.0001
Ether extract	3.55^a^	1.39^c^	1.69^c^	1.38^c^	2.71^b^	2.93^ab^	0.18	<0.0001
Nitrogen-free extract	49.69^b^	50.11^ab^	52.3^a^	48.73^b^	50.54^ab^	50.67^ab^	0.35	0.0369
Total digestibility of nutrient	49.9^a^	43.39^c^	46.65^b^	42.14^c^	50.32^a^	49.4^a^	0.72	<0.0001
Calcium	15.83^a^	5.45^c^	5.89^c^	14.08^a^	14.62^a^	10.18^b^	0.96	<0.0001
Phosphor	4.47^a^	2.08^c^	2.09^c^	4.17^a^	4.34^a^	3.29^b^	0.23	<0.0001
Neutral detergent fibers	76.17^ab^	77.64^a^	74.29^bc^	76.02^ab^	71.92^d^	72.81^dc^	0.47	0.0002
Acid detergent fiber	63.32^c^	69.86^a^	67.42^b^	59.9^d^	53.92^e^	62.82^c^	1.03	<0.0001
Acid detergent lignin	15.71^b^	17.09^a^	15.83^b^	16.53^ab^	11.42^d^	13.62^c^	0.40	<0.0001
Cellulose	42.62^b^	47.76^a^	46.59^a^	38.37^c^	37.50^c^	44.20^b^	0.81	<0.0001
Hemicellulose	12.85^b^	7.78d^c^	6.87^d^	16.12^a^	18.00^a^	9.99^c^	0.88	<0.0001

Means in the same column with different superscripts differ significantly (p < 0.05). CsA = Citronella straw with ammoniation treatment, CsFP = Citronella straw fermented with *Pleurotus ostreatus*, CsFAn = Citronella straw fermented with *Aspergillus niger*, CsAFTh = Citronella straw with ammoniation treatment followed by fermentation with *Trichoderma harzianum*, CsAFP = Citronella straw with ammoniation treatment followed by fermentation with *Pleurotus niger*, CsAFAn = Citronella straw with ammoniation treatment followed by fermentation with *Aspergillus niger*

Fiber fractions (NDF, ADF, and ADL) exhibited a wide variation, reflecting structural carbohydrate modifications resulting from ammoniation and fungal fermentation, particularly through lignin degradation and partial hemicellulose solubilization.

- Fiber fractions: CsAFP and CsAFAn had the lowest NDF compared to CsA (control). CsAFP also showed the lowest ADF (53.9% vs. 63.3% in CsA) and ADL (11.4% vs. 15.2% in CsA)
- DM: CsFP and CsFAn recorded the highest DM values (88.8%–89.2%), whereas ammoniated treatments (CsA, CsAFTh, CsAFP, and CsAFAn) exhibited lower DM (~41–42%) due to increased moisture
- OM and ash: OM ranged from 90.4% to 92.4%, with CsAFTh showing the lowest value. Ash content peaked in CsAFTh (9.59%), suggesting enhanced mineral accumulation during fermentation with *T. harzianum*
- CP: CsAFP yielded the highest CP (9.1%), followed by CsA (8.4%) and CsAFAn (8.1%). In contrast, single fermentations with *P. ostreatus* and *A. niger*, or combined ammoniation-fermentation with *T. harzianum*, resulted in lower CP (6.4%–6.7%), suggesting that ammoniation elevated protein levels while fermentation alone caused slight degradation
- CF and EE: CsFP and CsAFTh produced higher CF, whereas CsAFP had the lowest CF (29.7%), indicating enhanced fiber degradation by *P. ostreatus* following ammoniation. The highest EE was recorded in CsA (3.5%), while single fermentation and CsAFTh treatments reduced EE (1.4%–1.7%), likely due to fungal lipase activity.

### Digestibility

Digestibility values (Table 2) revealed significant improvements (p < 0.01) across treatments.

**Table 2:** Digestibility of nutrients in the treatment with citronella straw (%Dry matter).

Nutrient digestibility (%)	Treatments						SEM	p-value

	CsA	CsFP	CsFAn	CsAFTh	CsAFP	CsAFAn		
Dry matter	43.68^c^	41.06^d^	40.54^d^	42.09^dc^	53.98^a^	51.64^b^	1.06	<0.0001
Organic matter	47.09^c^	44.01^d^	45.04^dc^	45.19^dc^	61.26^a^	55.08^b^	1.29	<0.0001
Neutral detergent fibers	31.38^c^	34.00^b^	32.19^c^	34.45^b^	37.24^a^	35.13^b^	0.42	<0.0001
Acid detergent fiber	37.24^c^	39.27^b^	37.66^c^	40.67^b^	46.49^a^	36.89^c^	0.67	<0.0001
Acid detergent lignin	28.69^bc^	24.13^d^	32.52^ab^	33.89^a^	30.38^bac^	28.30^c^	0.78	0.0003
Cellulose	23.53^c^	29.84^b^	25.2^c^	25.32^c^	33.47^a^	29.87^b^	0.72	<0.0001
Hemicellulose	30.84^bc^	33.47^a^	31.65^b^	33.91^a^	29.71^c^	30.45^bc^	0.36	<0.0001

Means in the same column with different superscripts differ significantly (p < 0.05). SEM = Standard error of the mean, CsA = Citronella straw with ammoniation treatment, CsFP = Citronella straw fermented with *Pleurotus ostreatus*, CsFAn = Citronella straw fermented with *Aspergillus niger*, CsAFTh = Citronella straw with ammoniation treatment followed by fermentation with *Trichoderma harzianum*, CsAFP = Citronella straw with ammoniation treatment followed by fermentation with *Pleurotus niger*, CsAFAn = Citronella straw with ammoniation treatment followed by fermentation with *Aspergillus niger*

- DM and OM digestibility: CsAFP and CsAFAn had the highest DMD and OMD, whereas CsFP and CsFAn had the lowest.
- Fiber digestibility: NDFD, ADFD, and cellulose digestibility were highest in CsAFP and CsAFAn, indicating superior fiber utilization. ADL digestibility was greatest in CsFAn and CsAFTh. Hemicellulose digestibility increased in CsAFTh and CsFP but was lowest in CsAFP.
- Cell wall reduction: Cellulose content was lowest in CsAFTh (38.4%) and CsAFP (37.5%) compared with control CsA (42.6%). Hemicellulose content was highest in CsAFTh (16.1%) and CsAFP (18.0%) versus CsA (12.8%).

Overall, ammoniation combined with *P. ostreatus* or *A. niger* fermentation improved the digestibility of structural carbohydrates, whereas hemicellulose remained less digestible due to strong lignin cross-linkages.

### Ruminal fermentation

Treatment effects on ruminal fermentation were highly significant (p < 0.01) for NH_3_ concentration (p < 0.0001), total VFA (p = 0.0216), acetate (p = 0.0376), propionate (p = 0.0108), iso-butyrate (p = 0.0015), iso-valerate (p < 0.0001), acetate-to-propionate ratio (p = 0.0118), and CH) production (p = 0.0149).

- NH**_3_**: Highest in CsA; reduced in CsFP, CsFAn, and CsAFAn.
- VFAs: Total VFA peaked in CsA and was lowest in CsAFAn. Acetate was highest in CsFP (73.9 mM) and lowest in CsAFAn (70.6 mM). Propionate was highest in CsAFP and CsAFAn, lowering the A:P ratio in these groups.
- CH**_4_**: CsAFAn reduced CH_4_ output by 0.8 mmol/L compared to CsA, indicating enhanced fermentation efficiency.
- Stable parameters: pH, n-butyrate, and n-valerate were unaffected (p > 0.05), showing buffering capacity of the rumen environment.

### Phenolic content

The total phenolic content (expressed as gallic acid equivalents) was highest in ammoniated-fermented treatments (CsAFP and CsAFAn, 4.3%). Other treatments ranged from 3.19% to 3.9%. Ammoniation released phenolic compounds, while fungal ligninolytic enzymes further enhanced availability. In contrast, single fermentation (CsFP, CsFAn) reduced detectable phenols, possibly due to transformation into other forms.

### Multivariate treatment effects

**Figure 1:**
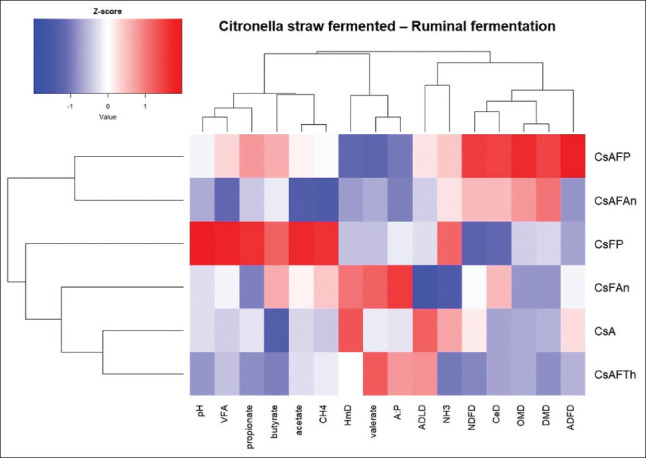
Heatmap of the relationship between treatments and *in vitro* rumen fermentation characteristics of citronella straw colors reflect a trend of treatment effects from a decreasing trend (blue) to an increasing trend (red). DMD = Dry matter digestibility, OMD = Organic matter digestibility, NDFD = Neutral detergent fiber digestibility, ADFD = Acid detergent fiber digestibility, HmD = Hemicellulose digestibility, CeD = Cellulose digestibility, ADLD = Acid detergent lignin digestibility, A:P = Ratio of acetic acid to propionic acid, CsA = Citronella straw with ammoniation treatment, CsFP = Citronella straw fermented with *Pleurotus ostreatu*s, CsFAn = Citronella straw fermented with *Aspergillus niger*, CsAFTh = Citronella straw with ammoniation treatment followed by fermentation with *Trichoderma harzianum*, CsAFP = Citronella straw with ammoniation treatment followed by fermentation with *Pleurotus niger*, CsAFAn = Citronella straw with ammoniation treatment followed by fermentation with *Aspergillus niger*.

Cluster analysis (Figure 1) revealed distinct groupings. CsAFP and CsFAn formed one cluster, reflecting similar effects on fermentation. CsAFTh and CsFAn treatments grouped closely with *P. ostreatus*-fermented straw, while CsA was distinct, indicating divergent effects. Color gradients (blue–red) illustrated treatment impact intensity across parameters, aiding identification of optimal strategies for improving fermentation efficiency and reducing undesirable outputs.

### Palatability

**Figure 2:**
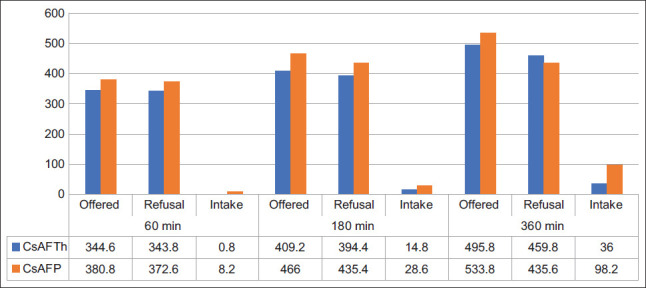
Voluntary feed intake (g) of citronella straw ammoniation-fermented *Trichoderma harzianum* (CsAFTh) and *Pleurotus ostreatus* (CAFP) at 60, 180, and 360 min post-feeding.

The palatability test compared CsAFTh and CsAFP in Ettawa goats. VFI was recorded at 60, 180, and 360 min (Figure 2).

- At 60 min, CsAFTh intake was minimal (0.8 g), while CsAFP was higher (8.2 g)
- At 180 min, CsAFTh and CsAFP intake increased to 14.8 g and 28.6 g, respectively
- At 360 min, CsAFP intake reached 98.2 g, markedly higher than CsAFTh (36 g).

Overall, CsAFP consistently showed superior palatability and acceptance compared with CsAFTh, confirming its potential as a more practical feeding strategy.

### Summary of key findings

Overall, the combination of ammoniation and fungal fermentation substantially altered the nutritional quality, digestibility, and fermentation profile of citronella straw. Among treatments, CsAFP (ammoniation + *P. ostreatus*) consistently produced the most favorable outcomes, including reduced fiber fractions (NDF, ADF, and ADL), improved CP content, enhanced DM and OM digestibility, increased propionate levels, and significantly higher voluntary feed intake. CsAFAn (ammoniation + *A. niger*) also performed well, particularly in improving digestibility and reducing CH_4_ emissions. In contrast, single fermentation treatments (CsFP and CsFAn) yielded modest improvements, while ammoniation alone (CsA) showed limited effects. These results collectively indicate that integrated chemical–biological treatment, particularly with *P. ostreatus*, offers the greatest potential for upgrading citronella straw into a sustainable and palatable ruminant feed resource.

## DISCUSSION

### Chemical composition

The differences in the chemical composition of citronella straw among the treatments can be explained by the effects of fungal fermentation and ammoniation, both of which play key roles in altering fiber structures, nutrient availability, and overall composition. The highly significant variations suggest that fermentation affected moisture dynamics, with higher DM content observed in fungal-treated groups (CsFP, CsFAn), likely due to microbial metabolism reducing moisture through the breakdown of structural carbohydrates, similar to Tuyen *et al*. [23]. The OM and ash content also varied significantly, possibly due to microbial activity modifying mineral profiles, resulting in slight changes in ash levels. This trend is consistent with observations in fungal-treated rice straw, where *Pleurotus* species influenced ash content by altering mineral bioavailability [10]. Both ammoniation and fungal action affect CP levels; ammoniation increases nitrogen content, thereby enhancing CP [3, 8], while fungal fermentation [19] may either utilize existing protein or incorporate nitrogen into fungal biomass. Significant changes in CF, NDF, and ADF reflect the degradation of lignocellulosic structures by fungi, such as *Pleurotus* and *Aspergillus*, which are known to break down lignin and facilitate fiber digestibility [24]. The enhanced breakdown of hemicellulose and cellulose [25] contributes to improved microbial access to nutrients. The EE content was lower in fungal-treated groups, indicating lipid degradation by microbial enzymes, consistent with findings where fungal treatments reduced the fat content in various feedstuffs [11]. Among all treatments, CsAFTh significantly increased CP while decreasing fiber components, highlighting the synergistic benefits of combining ammoniation with fungal fermentation [3] to improve the nutritive value and digestibility of citronella straw. Similarly, a previous study by Wang *et al*. [26] showed that fermenting highland barley straw with *P. ostreatus* significantly increased CP content from 4.51% to 5.46% compared with the control. *P. ostreatus* decomposes organic substrates to acquire C and N, which are essential for growth and development. The observed increases in TDN and NFE suggest enhanced carbohydrate digestibility and availability, likely due to fungal degradation of fibrous components into simpler, more accessible sugars [12]. Fungi such as *Trichoderma* and *Aspergillus* [16] use enzymatic hydrolysis to break down lignocellulose, thereby increasing the proportion of fermentable carbohydrates and improving the feed’s overall energy content. Ammoniation was found to influence the solubility of Ca and P, with significant differences attributed to fungal absorption and metabolic activity. Similar to the findings in fungus-treated rice straw, microbial fermentation [17] appears to modify the bioavailability of mineral profiles. Low levels of NDF, ADF, ADL, and cellulose, paired with high hemicellulose content, are beneficial for improving feed digestion and overall digestibility. Reduced NDF reflects a lower amount of total fiber, which helps minimize rumen fill and supports greater voluntary feed intake in ruminants. Similarly, a decrease in ADF indicates lower cellulose and lignin concentrations, enhancing fiber digestibility compared to feeds with higher fiber content. Because ADF is closely associated with the least digestible portions of plant material, mainly cellulose and lignin, its increase typically results in reduced digestibility and energy yield for forage [27]. A lower ADL value also indicates decreased lignin, which is a major barrier to digestion, thereby enabling rumen microbes to break down plant fibers more efficiently and generate VFA-derived energy. This study is in line with a previous study by Wang *et al*. [26], which reported that highland barley straw fermented with *P. ostreatus* significantly decreased NDF (64.97% to 47.94%), ADF (39.83% to 31.55%), and ADL (19.01% to 10.21%) compared with the control.

### Phenolic content

**Figure 3:**
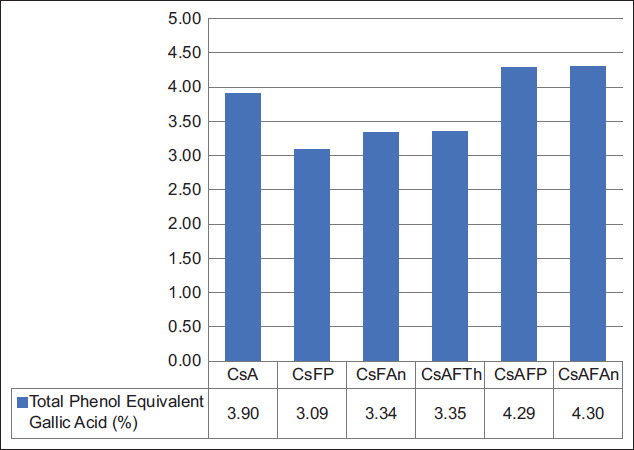
Total phenol equivalent to gallic acid in the treatment of citronella straw.

The total phenol content (as gallic acid equivalents) exhibited lower concentrations in comparison to the total phenol content found in the *Quercus robur* L. extract as documented by Formato *et al*. [28], which was approximately 200–800 mg GAEs/g extract, or about 20%–80% of the extract used as an antioxidant source. Nevertheless, the total phenol content remaining in the citronella straw is suspected to have the potential as an antioxidant compound and could optimize digestion and fermentation in the rumen. Fungus is well known to modulate the lignocellulosic properties of high-fiber substrates during solid-state fermentation (SSF). Figure 3 shows that ammoniated and fermented citronella straw with *Pleurotus ostreatus* and *A. niger* tended to produce high total phenol content compared with other treatments. This indicates that the ammoniation and fermentation process using fungus provides optimal results in enhancing the availability of phenolic compounds in citronella straw due to microbial activity in lignin degradation. Agro-industrial residues, such as citronella straw, contain a high fraction of CF, particularly lignin. The lignin fraction binds several phenolic compounds, such as ferulic, coumaric, syringic, and hydroxybenzoic acids, which can be extracted through fermentation [29–31]. Fungus will secrete β-xylosidase, β-galactosidase, α-amylases, and cellulase enzymes during SSF to degrade the complexity of lignin and release bioactive compounds, such as total phenol. The substrates can induce the production of different extracellular enzymes from the fungus, which can act on the lignocellulosic material and enhance the release of bioactive compounds. The fungus that grows during the fermentation process of citronella straw will use polysaccharides for their growth after the lignin degradation process [32, 33]. A previous study by Schmidt *et al*. [29] showed an increase in the total phenol concentration in rice bran from 2.4 mg/g to 5.1 mg/g after fermenting for 120 h with the fungus *Rhizopus oryzae*. The successful increment of total phenol availability was also reported by Pinela *et al*. [32], who evaluated brewer’s spent grain fermented with *R. oryzae* and by Purewal *et al*. [34], who fermented barley with *Aspergillus* *awamori* during SSF. GAE increased from 25.2 mg to 58.1 mg/mg during SSF of oat bran fermented with *A. niger* for 6 days [35]. This microbe has two different extracellular systems: One break down carbohydrates by synthesizing them and the other is a ligninolytic oxidative system that breaks down phenyl rings. This procedure increases the amount of free phenolic compounds present in the substrate. *P. ostreatus*has potential as an antioxidant agent due to its phenolic content, so that it plays a role in increasing total phenol in the recent study. A previous study by Egra *et al*. [36] reported that *Pleurotus* spp. extracted with n-hexane resulted in 78.5 mg GAE/g of phenolic compound. Phenolic compounds released during lignocellulose degradation contribute to antioxidant activity and rumen modulation. However, excessive phenolic content has been reported to impair nutrient digestibility and reduce palatability at certain levels. As previous studies by Jalal *et al*. [37] and Agustinho *et al*. [38] have reported, the total phenolic content of 243.8 mg GAE/g was found to reduce OM digestibility. Conversely, a previous study by Pinela *et al*. [32] reported that dikaryotic and monokaryotic strains of *Pleurotus sapidus* for SSF reduced the total phenol content of the substrates of sunflower seed hulls, rice husks, and rice straw. Several factors, such as the content of lignin in the substrate, the fungus species, the genotype by environmental interaction, the fermentation times, and culture conditions, can influence the production and availability of bioactive compounds in the substrate.

### Digestibility

Degradation of lignin by *Pleurotus* and *Aspergillus* species enhances fiber digestibility. *P. ostreatus* produces lignin-degrading enzymes, such as lignin peroxidase (LiP), MnP, and Lac, which reduce lignin content and improve digestibility [38–40]. A previous study by Agustinho *et al*. [38] reported the enzyme activity of LiP, MnP, and Lac in *P. ostreatus*, namely 176, 12.6, and 390 U/g, respectively, using a ultraviolet-visible spectrophotometer. However, the balance between lignin degradation and nutrient retention varies with fungal species and fermentation conditions [41]. Machado *et al*. [42] also reported an increase in digestibility in sugarcane silage using an enzymatic complex (U/min/g of enzymatic complex) produced by a white rot fungus, with the following activities: Lac (191.3), MnP (15.4), LiP (12.6), carboxymethylcellulase (206.2), mannanase (111.1), and xylanase (202.0). *Pleurotus* (white-rot fungi) and *Trichoderma* species efficiently degrade lignocellulose by producing ligninolytic enzymes, including Lac, MnP, and LiP [40, 43–45]. These enzymes break down lignin and hemicellulose, making cellulose more accessible and reducing ADF and ADL levels in treated biomass. ADF consists mainly of cellulose and lignin, whereas ADL represents the most recalcitrant fraction of lignin. The significant reduction in ADF and ADL suggests that CsAFP treatment effectively broke down rigid fiber structures, improving digestibility [46]. White-rot fungi, particularly *P. estrus* species, selectively degrade lignin [47] more efficiently than cellulose, reducing ADL levels while preserving digestible fibers. This selectivity improves fiber digestibility and minimizes nutrient loss. This study corroborates the findings of Agustinho *et al*. [38], who fermented whole-plant corn with *P. ostreatus* and observed greater *in vitro* digestibility (61.3%) than untreated plant corn (56.3%). They stated that the increase in *in vitro* digestibility depends on the modification of the chemical composition of whole plant corn due to the reduction in lignin and cellulose composition. The specific composition of hemicellulose in feed ingredients can affect its availability to rumen microbes. The high digestibility of NDF and ADF indicates that most of the fiber components can be well used by rumen microbes. Furthermore, the high digestibility of cellulose indicates that the main structural fiber fraction can be efficiently degraded. However, the low digestibility of hemicellulose can inhibit the total energy utilization of the feed, especially if hemicellulose is a significant fiber component. Feed with high digestibility in DM and OM will increase energy availability for animals, supporting milk production and meat growth. However, the low digestibility of hemicellulose can affect the overall effectiveness of fiber utilization, especially in forage-based feed systems with high hemicellulose content. However, the high content of hemicellulose still has to be confirmed in the digestibility coefficient of ruminant livestock, either through *in vitro* or *in vivo* tests, because sometimes there is still a cross-linking of hemicellulose with high ADL, and it is difficult to hydrolyze. Fibrolytic enzymes, including those that specifically target hemicellulose, break it down into simpler sugars that can be utilized by ruminants [48]. Enzymes can be applied in different forms (liquid or granular) to different feed types, including hay, silage, and total mixed rations. The use of exogenous enzymes has been linked to increased milk and meat production, as they improve feed digestibility [49].

### Ruminal fermentation

The highest NH_3_ concentration in CsA suggests that NH_3_ increases nitrogen availability by breaking down fiber-bound nitrogen [50]. Lower NH_3_ in CsFP, CsFAn, and CsAFAn could be due to microbial assimilation of NH_3_ during fungal fermentation [51]. However, NH_3_ inhibition poses a significant challenge [52] to the efficient and stable operation of anerobic digestion systems by inhibiting VFA conversion and reducing CH_4_ production. A higher total VFA in CsA indicates enhanced fermentation activity, likely due to improved fiber digestibility from ammoniation [53]. The lower VFA in CsAFAn suggests that fungal fermentation altered the fiber structure, possibly favoring the growth of selective microbial populations [54]. Numerous forage types, including those fermented by fungi, such as *A. niger*, have been shown to enhance rumen fermentation parameters, such as the production of VFAs, which are essential for ruminant energy metabolism [55, 56]. This supports the results of Rabee *et al*. [57], who found that dietary changes can significantly impact microbial activity and fermentation efficiency. Similarly, Zhao *et al*. [58] reported a high potency of *P. ostreatus* fermentation of corn straw to optimize VFA production up to 77.45 mmol/L compared with the control (70.49 mmol/L). The increase in acetate content is likely due to fungal fermentation causing lignocellulose degradation [59]. The higher propionate content in CsAFP and CsAFAn suggests enhanced microbial pathways that favor propionate over acetate production, thereby reducing the A:P ratio and leading to improved energy use [60]. This finding aligns with a previous study of Wang *et al*. [61], who reported an increase in propionate on rice straw treated with *P. ostreatus*, resulting in a reduction of the A:P ratio (2.16) compared to untreated rice straw (3.21). The lower CH_4_ content in CsAFAn is likely due to a shift in fermentation that favors propionate production, which competes with methanogenesis for hydrogen [62]. Fungal-treated feeds may also contain bioactive compounds that suppress the growth of methanogenic archaea [63–65]. Similar to a previous study that reported that sorghum treated with *P. ostreatus* reduced CH_4_ production in the rumen. This can be attributed to improved digestibility, and the propionate production pathway may have enhanced hydrogen utilization, thereby acting as an H_2_ sink and reducing CH_4_ formation during enteric fermentation [66]. The non-significant pH differences suggest that despite fermentation variations, buffering mechanisms in the rumen maintained pH stability [61]. Isobutyrate and isovalerate variations may result from differences in fungal treatment-induced protein degradation pathways [67].

### Treatment impacts

As indicated in the heatmap visualization, CsAFP resulted in the highest digestibility (DMD, OMD, NDFD, ADFD, and CeD), followed by CsAFAn. Both CsAFP and CsAFAn treatments tended to decrease acetate acid levels and the acetate-to-propionate ratio (A:P), while increasing total VFA and propionate and butyrate levels. However, treatments involving fermentation with microbes tended to decrease CH_4_ production. Fungal fermentation enhances fiber degradation by producing ligninolytic enzymes, thereby improving digestibility metrics. Treatment of oil palm frond-based feed silage with cellulase enzymes significantly increased *in vitro* nutrient digestibility. The heatmap analysis revealed notable differences in the ruminal fermentation profiles among the various fermentation treatments. Treatments, such as CsAFP and CsAFAn, were associated with higher nutrient digestibility (DMD, NDFD, and CPD) and greater VFA production, suggesting improved fermentation efficiency. These treatments also showed stronger positive correlations with acetate, propionate, and butyrate levels, which are key indicators of energy-yielding fermentation pathways [68]. In contrast, CsFAn displayed more negative correlations across most parameters, indicating lower fermentative activity. The relatively lower CH_4_ values observed in some treatments (CsAFP) suggest the potential for CH_4_ mitigation, which is an important consideration for sustainable ruminant nutrition [69]. The clustering pattern further supports the similarity of effects within treatment groups [70, 71], highlighting the influence of fermentation type or additive on rumen microbial activity and feed utilization.

### Palatability

Palatability testing is a key method for assessing the willingness of animals to consume a particular diet, which is crucial for ensuring sufficient nutrient intake and overall performance. The fermentation advantages of *A. niger* in animal feed are described by Wang *et al*. [55], who also emphasize its improvement of nutritional availability and palatability. In this study, VFI in goats exhibited a time-dependent increase, with intake rising from approximately 8.2 g at 60 min to 98.2 g at 360 min. Despite the consistent quantities of feed being offered, high levels of refusal indicated selective feeding behavior. Notably, goats consistently consumed more of the CsAFP diet than CsAFTh across all time intervals, suggesting a preference likely influenced by palatability attributes, such as texture, aroma, and moisture content. Behavioral factors, such as exploratory feeding and adaptation over time, may have further contributed to these patterns. These findings support previous reports highlighting the importance of feed composition and exposure duration in influencing VFI [71]. The role of palatability in ruminant nutrition is well-documented, as animals exhibit sensitivity to feed characteristics, including smell and taste, which are influenced by feed formulation, processing methods, and ingredient freshness [69, 70]. Therefore, the observed differences in VFI between the CsAFP and CsAFTh treatments can be attributed to variations in palatability and nutrient composition, with the CsAFP diet being more readily accepted by the goats. *Pleurotus* spp. secrete LacMnP and LiP that cleave and demethylate lignin oxidatively, cleave ether and C–C bonds, and break ester bridges between lignin and hemicellulose. Reduced ADL/ADF, enhanced digestibility, and VFA shift to propionate (reduced A:P) – propionate rises as more hemicellulose/cellulose is released and hydrogen in fermentation is shunted away from methanogenesis. Recent *in vitro* studies on *P. ostreatus*-treated corn stover showed that total VFAs increased, C3 increased, and A:P decreased, replicating Table 3 trends and validating the CH_4_ reduction (p = 0.0149) [72]. As part of fungal pre-treatment, these oxidative enzymes (Lac, MnP, LiP) are uniformly referenced [73] as the principal lignin deconstruction inducers that precede carbohydrate hydrolysis. *A. niger* contributes high activities of cellulases, xylanases, β-glucosidases, pectinases, and esterases that degrade lignin–carbohydrate esters. In this study, such activity reduced cellulose/hemicellulose, increased TDN, and enhanced DM/OM digestibility (p < 0.0001), with a moderate impact on ADL unless combined with *Pleurotus* or ammoniation (oxidation + hydrolysis). The low-cost manufacture of *A. niger* enzyme consortia on agro-residues is well established and feasible for smallholder systems [74, 75]. Its cellulases are widely used to saccharify rice straw after mild alkali/acid pre-treatment, an enzyme cascade parallel to the CsAFAn improvements [76]. *T. harzianum* and *Trichoderma reesei* are also pre-eminent in cellulase/xylanase secretion and can support oxidative enzymes. *Pleurotus* “opens” lignin, and *Trichoderma* rapidly depolymerizes exposed polysaccharides, explaining the synergy observed in ammoniation + fermentation treatments, which showed large increases in DM/OM/NDF/ADF digestibility (p < 0.0001). Enzyme-omics studies reveal that *Trichoderma* adjusts its cellulase/xylanase composition in response to pre-treated straws, accounting for the dramatic drops in NDF/ADF and improved digestibility [77].

**Table 3:** *In vitro* ruminal fermentation characteristics after treatment with citronella straw.

Parameter	Treatments						SEM	p-value

	CsA	CsFP	CsFAn	CsAFTh	CsAFP	CsAFAn		
Ammonia (mM)	7.73^a^	4.50^c^	5.01^c^	7.16^ab^	6.80^b^	6.53^b^	0.25	<0.0001
pH	6.96	6.72	6.66	6.72	6.74	6.68	0.04	0.3732
Total VFA (mM)	10.26^a^	8.24^b^	8.30^b^	8.37^b^	8.66^b^	7.56^b^	0.24	0.0216
Partial VFA (%)								
Acetate (C_2_)	72.17^abc^	73.93^a^	73.45^ab^	72.38^abc^	70.98^bc^	70.63^c^	0.36	0.0376
Propionate (C_3_)	24.40^ab^	22.26^b^	23.28^b^	24.56^ab^	25.42^a^	25.55^a^	0.31	0.0108
Iso-butyrate (_I_C_4_)	0.28^c^	0.33^ab^	0.35^ab^	0.32^b^	0.32^ab^	0.36^a^	0.01	0.0015
N-butyrate (_n_C_4_)	2.91	3.15	3.22	3.06	3.04	3.16	0.05	0.6017
Iso-valerate (_I_C_5_)	0.16^b^	0.24^a^	0.24^a^	0.21^a^	0.17^b^	0.21^a^	0.01	<0.0001
N-valerate (_n_C_5_)	0.08	0.09	0.10	0.08	0.07	0.08	0.00	0.0575
A: P	2.97^ab^	3.33^a^	3.17^a^	2.95^b^	2.79^b^	2.79^b^	0.05	0.0118
CH_4_ production (mmol/l)	2.79^a^	2.33^b^	2.31^b^	2.25^b^	2.28^b^	1.95^b^	0.07	0.0149

Means in the same column with different superscripts differ significantly (p < 0.05). VFA = Volatile fatty acid, SEM = Standard error of the mean, CsA = Citronella straw with ammoniation treatment, CsFP = Citronella straw fermented with *Pleurotus ostreatus*, CsFAn = Citronella straw fermented with *Aspergillus niger*, CsAFTh = Citronella straw with ammoniation treatment followed by fermentation with *Trichoderma harzianum*, CsAFP = Citronella straw with ammoniation treatment followed by fermentation with *Pleurotus niger*, CsAFAn = Citronella straw with ammoniation treatment followed by fermentation with *Aspergillus niger*

## CONCLUSION

This study demonstrated that the combination of ammoniation and fungal fermentation significantly improved the nutritional value, digestibility, and utilization efficiency of citronella straw. Among all treatments, CsAFP (ammoniation + *P. ostreatus*) consistently showed superior performance, with the highest CP (9.1%), the lowest fiber fractions (NDF, ADF, ADL), enhanced DM and OM digestibility, increased propionate levels, and the highest voluntary feed intake in goats. CsAFAn (ammoniation + *A. niger*) also showed favorable outcomes, particularly in improving fiber digestibility and reducing CH_4_ emissions, while single fermentation treatments (CsFP and CsFAn) produced moderate effects. Ammoniation alone (CsA) improved nitrogen availability but was less effective in reducing fiber fractions or improving palatability.

The findings highlight the potential of citronella straw, a widely available agro-industrial byproduct in Indonesia and other citronella-producing regions, as a cost-effective alternative feed resource for ruminants. Upgrading this biomass through ammoniation–fungal fermentation can reduce farmers’ reliance on costly concentrate feeds, increase feed efficiency, and support sustainable livestock production. Furthermore, the observed reduction in CH_4_ emissions, particularly with CsAFAn, suggests an important environmental co-benefit that aligns with climate change mitigation goals.

This work has several strengths, including being the first systematic evaluation of citronella straw upgrading using chemical–biological treatments, testing three fungi with different lignocellulolytic capacities, and integrating chemical, nutritional, fermentation, and behavioral parameters to offer a comprehensive assessment. It also demonstrates the practical value of a low-cost and locally adaptable method relevant for smallholder farmers. However, the study was limited by reliance on *in vitro* assays, short-term palatability trials, and the lack of long-term performance data on growth or milk yield. The potential anti-nutritional effects of residual phenolic compounds have not been fully evaluated, and fermentation outcomes may vary depending on substrate composition, fungal strains, and culture conditions.

Future research should focus on *in vivo* feeding trials to validate digestibility, performance, and CH_4_ reduction under practical farm conditions, alongside studies on enzyme activity profiles, long-term effects on rumen microbiota and health, economic feasibility, and controlled release of phenolic compounds.

In conclusion, ammoniation–fungal fermentation, particularly with *P. ostreatus*, is a robust and practical method for upgrading citronella straw into a valuable ruminant feed resource. The integrated approach not only enhances nutrient availability and palatability but also offers a pathway for waste valorization and CH_4_ mitigation. By bridging laboratory findings with field relevance, this study contributes to sustainable livestock production in citronella-producing regions and lays the groundwork for future innovations in feed biotechnology.

## AUTHORS’ CONTRIBUTIONS

DP, YY, SS, GET, IH, and BA: Conceptualized the study, methodology, and manuscript preparation. MM, WN, ASM, EMP, and PSN: Data collection, methodology, and manuscript preparation. PCP, KS, ZE, and DSW: Methodology, formal analysis, and drafted the manuscript. All authors have read and approved the final manuscript.
